# Horses’ Response to a Novel Diet: Different Herbs Added to Dry, Wet or Wet-Sweetened Oats

**DOI:** 10.3390/ani12111334

**Published:** 2022-05-24

**Authors:** Anna Stachurska, Ewelina Tkaczyk, Monika Różańska-Boczula, Wiktoria Janicka, Iwona Janczarek

**Affiliations:** 1 Department of Horse Breeding and Use, Faculty of Animal Sciences and Bioeconomy, University of Life Sciences in Lublin, 20-950 Lublin, Poland; anna.stachurska@up.lublin.pl (A.S.); wiktoria.janicka@up.lublin.pl (W.J.); iwona.janczarek@up.lublin.pl (I.J.); 2 Department of Applied Mathematics and Computer Science, Faculty of Production Engineering, University of Life Sciences in Lublin, 20-950 Lublin, Poland; monika.boczula@up.lublin.pl

**Keywords:** horse, herb, feed, taste, smell, palatability, welfare

## Abstract

**Simple Summary:**

The commercial horse feed industry uses palatants to mask undesirable tastes of feed and enhance consumption. However, an unknown smell or taste may also hinder feed intake, due to, among other aspects, novelty. The acceptability of herbs by horses has not been studied. We analysed whether five herbs (field mint, common yarrow, common chamomile, common sage and common nettle) added alternately to oats and presented within a dry, wet or wet-sweetened diet influence horses’ willingness to consume. Twenty horses were given different diet combinations of a feed presentation and a herb consecutively, once daily. Seven parameters showing the willingness to consume were measured: times of olfaction and consumption, times and numbers of intervals in consumption and drinking water, and mass of leftovers. The results show that the herbs in the amount offered did not influence the time of intake and only the dry oats with common sage added were smelled longer before consuming. However, wetting or wetting and sweetening the feed increased the willingness to eat. In conclusion, herbs in small amounts do not affect the feed intake, whereas wetting and sweetening the diet is of great importance and should be regarded when preparing horse diets.

**Abstract:**

The commercial horse feed industry uses palatants to mask undesirable tastes of feeds and enhance product acceptance. However, an unknown odour or taste may also hinder feed intake, due to, inter alia, novelty. The objective of the study was to assess the horses’ response to novel diet: five different herbs added alternately to dry, wet or wet-sweetened oats. Twenty adult horses were given different diet combinations of a feed presentation and a herb: field mint, common yarrow, common chamomile, common sage and common nettle, consecutively, once daily. The response to novelty was assessed regarding traits showing the willingness to consume: times of olfaction and consumption, times and numbers of intervals in consumption and drinking water, and the mass of leftovers. The results show that properties of the herbs studied did not hinder the consumption and only the odour of the dry common sage delayed the intake. Wetting or wetting and sweetening the diet accelerated the intake. In conclusion, herbs in small amounts do not significantly affect the willingness to consume feed. Although wet and wet-sweetened diet presentations may be novel to horses, they increase the feed palatability and can be suggested for use when preparing horse diets.

## 1. Introduction

There are a lot of studies on feeding horses with a view to improving diets affecting the welfare of these animals managed under domestic conditions. Horses in nature may select and consume favourite plants, and avoid those which are unpalatable, indigestible or poisonous [1]. Horses have evolved a patch foraging strategy to select an appropriate diet from heterogeneous resources [2]. Such natural patch foraging behaviour also occurs in stabled horses when they are offered a variety of feeds. The horses’ selection relies on determining the odour, taste and texture of the feed [3]. The size and duration of a meal are regulated based primarily on the degree of hunger the horse was experiencing when it started to eat [4]. Not all horses reject poisonous plants and not always, which has been studied exemplarily concerning *Senecio jacobaea* L. [5,6].

Ranges and capacities of sensory abilities in humans and horses differ, hence, horses perceive their surroundings in a different way to humans [7]. Francis et al. [8] stated that equine preferences of treats tend to be based heavily on olfaction and gustation, whereas the human evaluation of products relies mainly on visual cues. However, according to Culda and Stermin [9], vision is horses’ main sense in the location of feed, followed by olfaction and taste; the latter contributes less to the selection. There are few studies on the role of senses during consumption by horses. It seems that horses have a well-developed sense of taste, similar to other herbivorous animals [10]. It is not known whether horses are able to associate odour and taste and form a concept of flavour in the same way as humans [7].

Some studies focus on horses’ taste and odour preferences [11]. Van den Berg et al. [12] found a preference for sweet aromatic flavours (banana and coconut) in horses. According to Randall et al. [13], foals prefer a sweet taste when the sucrose concentrations range from 1.25 to 10 g/100 mL. Salty, sour and bitter solutions resulted in indifference up to concentrations over which the foals’ response changed to rejection. The foals were quite variable in their response to a particular test chemical, for example, some of them avoided the taste of sucrose. Studies by Janczarek et al. [10] revealed that differences in taste preferences occur not only between individual horses but also between breeds and sexes. Purebred Arabian horses, for example, compared with Anglo-Arabian, Polish Konik and Polish Cold-Blooded horses were the most willing to consume sour apple and carrot pellets, whereas an addition of molasses was perceived as being tastier for mares than stallions. According to Provenza and Balph [14], the age of a horse also affects the feed preferences, since foraging experiences of young herbivores affect their dietary habits as adults.

There are homologies in the taste behavioural reactivity of humans, nonhuman primates and other species, including mice, rats, domestic cats and chicks [15,16,17,18]. Jankunis and Whishaw [19] found that the response to sweet and bitter substances in horses is also homologous to those species. Sucrose offered to horses elicited a bob coupled with a slight tongue protrusion and forward movement of ears, whereas bitter quinine caused a head extension and mouth gape accompanied by a large tongue protrusion and backward movement of the ears.

Palatants may be added to feed and medications to increase the acceptability of feed products by horses [20]. Various flavours mask undesirable taste and reduce neophobia. Hence, they are commonly used by the commercial horse-feed industry with the objective of improving intake [11,21]. The role of culinary herbs used in the human diet has been known for millennia. As a result of the enrichment of feeds with diverse odours and flavours, the feeding behaviours of horses may change, the feed nutritional value may increase and thus the horses’ welfare may be improved [22]. Goodwin et al. [3] found that providing variety in concentrate diets by manipulating sensory characteristics, for example, flavour, increases the nutritional variety whilst minimising the undesirable digestive consequences of changing the concentration ration and the hindering monotony of the diet.

Herbs not only influence the consumption patterns, secretion of digestive fluids and total feed intake in animals but also have many therapeutic properties. These effects in humans, long known in ancient civilisations, are nowadays scientifically documented. Naturally occurring herbs are particularly valuable compared with chemical compounds added to feed [23]. On the other hand, the usual strong odour and bitter taste of herbs may elicit neophobia in animals [24]. Neophobia has been defined as the fearful reaction to novel stimuli or situations, hence neophobic animals are less likely to consume novel food [25]. Van den Berg et al. [26] proved that a familiar odour increases the acceptance of a novel food, whereas novel odours may elicit neophobia in horses.

Little is known about the effect of herbs which may be used as palatants or supplements in the horse feed industry, for example, field mint (*Mentha arvensis* L.), common yarrow (*Achillea millefolium* L.), common chamomile (*Matricaria chamomilla* L.), common sage (*Salvia officinalis* L.) and common nettle (*Urtica dioica* L.) According to the knowledge of the anti-inflammatory and, in some cases, antioxidant properties of herbs applied in humans [27,28,29,30,31,32,33], these herbs are also used for horses. The desirable manner of feed presentation has not been studied either. Each horse examined in the present study was offered each kind of a novel feed only once, thus we considered the novelty effect instead of the neophobia associated with a repeated behaviour. We assumed that a herbal addition to oats presented in varying ways might positively or negatively influence the willingness to consume feed due to novelty. The objective of the study was to assess the horses’ response to a novel diet: five different herbs alternately added to dry, wet or wet-sweetened oats.

## 2. Materials and Methods

### 2.1. Horses

The study involved 20 adult (over five years old) warmblood horses. Their mean body weight was 520.2 ± 48.4 kg. The subjects were clinically sound, including their dental state. According to a veterinary physician and a caretaker who constantly took care of the horses, no symptoms of digestive system disorders had occurred for at least a year before the study. Two groups of seven horses and one group of six horses were kept in three stables under the same conditions during this time. They were maintained in individual stall boxes located at least four metres apart. The boxes were bedded with scobs, equipped with a crib and automatic waterer. The cribs were located on the box wall close to the stable corridor so that the feed could be poured into the crib directly from the corridor. The horses were fed three times a day at 6:00, 14:00 and 20:00, turned out for four hours daily and used for leisure riding for an hour daily six days a week. The diet for two months preceding the experiment contained 2.5 kg of oats and 8 kg of meadow hay, supplemented with 100 g of vitamin-mineral mixture (Platinum horse mineral, Sanowet, Poland). These amounts were determined according to requirements suggested by the National Research Institute for Agriculture, Food and the Environment (INRAE, France) and verified by observation of the horses’ actual body condition. The hay was divided into 2 kg for the morning, 2 kg for the afternoon and 4 kg for the evening ration. The afternoon hay ration was delivered at 13:00, i.e., one hour before the oats. The amount of oats was 1.0, 0.5 and 1.0 kg for the morning, afternoon and evening, respectively. None of the horses was given any herbs mixed with oats before the study, although they probably sometimes consumed the herbs in the pasture or in hay. The horses were never given wet or wet-sweetened everyday oats, hence the diets studied were novel.

### 2.2. Schedule of Offering Feeds

The effects of field mint, common yarrow, common chamomile, common sage and common nettle were analysed in the study. The common herbs were collected and packed by Podkowa AD 1905 (Lublin, Poland) and distributed by a store with products destined for horses in a dried-ground form. The herbal inclusion rate amounted to 10 g (3 g in the case of common sage). This amount, destined for one out of three daily meals, was 1/3 of the producer’s daily recommendations. It was mixed with 0.5 kg of oats with a wooden manual stirrer to obtain a dry feed, wetted with 100 mL of water to obtain a wet feed and additionally sweetened with 50 g of sucrose to obtain a wet-sweetened feed. Oats are a common feed given to horses in Poland and, as has been mentioned, the horses studied were also fed oats. For the sake of clarity, the feed (combination) of a given presentation (dry, wet or wet-sweetened) consisting of oats and a herb are henceforth named with two parts: the presentation and the herb, for example, dry field mint.

Feeds with a herbal supplement were given for the afternoon feeding when the hay ration was eaten. They were offered over three stages, each including six days, according to a modification of the Latin square method (three groups × three feed presentations × five herbs and the control; Table 1). The stages were divided with seven-day intervals, hence the entire experiment lasted 32 days. There was a control day at the beginning of each stage, when the oats of a given presentation without the herbal addition were offered and the horses were observed similarly to experimental days. Feeds of a different presentation for a particular stage were used in the three groups of horses. The feed presentation was not changed within a stage but the order of the herbs was altered within particular stages. Within a stage, a herb was added to the oats consecutively, one herb on one day in one stable, to prevent a mixing of the herb odours. Thus, each feed was novel and each horse was offered a given combination only once. The same herb combined with a novel manner of presentation was offered to a horse after more than eight days. Exemplarily, a horse from group 1 was offered dry oats on the first day (control) of the first experimental stage, then dry oats with the addition of one of the herbs on the next five days of the stage. After the seven-day interval, the wet oats were presented firstly without and then with the addition of a herb. Within the third stage, i.e., after the second interval, the wet-sweetened feed without or with the herbal addition was given. The total ration remained unchanged except for the herb addition.

### 2.3. Measurement of Data

The afternoon feed was delivered at the same time for all the horses in a stable. Each horse was studied by a familiar person. The observers stood motionless in the stable corridor, at a 1 m distance from each box. Each observer recorded the horse’s feeding behaviour on a video film and with a stopwatch. The observers were trained in how to record the variables before the study. Each time a horse began an action during the experiment, the observer clicked the stopwatch and simultaneously related the olfaction, consumption, interval or drinking to record the beginning of the action on the film. Based on the video film and stopwatch recordings, the following variables were determined:

(1) Time (s) of olfaction of the feed measured from the moment of approaching the crib until the moment of the beginning of consumption.
(2) Time (s) of consumption of the feed—total time of all moments of consumption, excluding intervals which lasted over 3 s.
(3) Time (s) of intervals in consumption—total time of all intervals in consumption which lasted over 3 s. Horses remained inactive during the intervals.
(4) Number of intervals in consumption.
(5) Time (s) of drinking water (irrespective of its amount)—total time of drinking from the beginning of consumption of the feed until 30 min after a horse had concluded consumption.
(6) Number of times of drinking water from the beginning of consumption of the feed until 30 min after a horse had stopped consumption.
(7) Mass of leftovers (g) which was taken away from the crib 30 min after the moment a horse had stopped consumption.

The methodology was determined based on a modification of earlier investigations conducted in horses [2,3,21] and our previous experience [10]. We assumed that a longer time of olfaction of a feed with an added herb meant that a horse concentrated on an unknown odour identification before ingestion which could make the animal abstain from consumption of the feed [34]. Reduced consumption times (time of consumption of a diet, time and number of intervals in the ingestion) and a low mass (or absence) of leftovers compared with the variables for the control diet show that a horse willingly consumed the feed. In turn, a long drinking time and frequent consumption of water indicates that a feed encourages the drinking of water [3,21].

### 2.4. Statistical Analysis

The measurements from all the horses studied were used in the analysis. The latter was performed utilising STATISTICA 13.3. The R software (R version 4.0.3) was used for some functions not available in Statistica. The normality of the data distribution was assessed with the Shapiro–Wilk test. The distribution of all the traits for feed presentation, herbs and combinations (feed presentation_herb) was not normal (*p* < 0.05). Transformation of the data did not give an expected effect, hence the non-parametric one-factor Friedman’s test for repeated measures was used. An initial analysis conducted with this method showed a significant influence of the feed presentation (*p* < 0.001) on all the variables studied. A similar analysis showed a significant effect of the herb on the variables (*p* < 0.001 for the time of olfaction, time of drinking water, number of times of drinking water and mass of leftovers; *p* < 0.05 for the time of consumption and number of intervals in consumption) except for the time of intervals in consumption (*p* = 0.06). The significant effects of the main experimental factors led us to compare 18 feed presentation_herb combinations using the Friedman’s test mentioned above. The multiple pairwise comparisons using the Nemenyi test were made as a post hoc analysis [35]. The variables are presented as mean values ± standard deviation (SD). A minimum level of significance was accepted at α = 0.05. The order of offering the feeds of a given presentation with a different herb in the horse groups was not taken into consideration, hence all the figures concern all 20 horses irrespective of the groups.

## 3. Results

**Figure 1:**
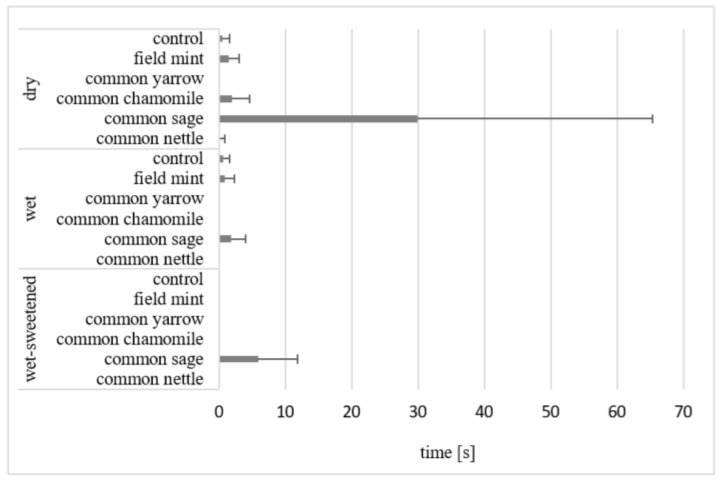
Mean time (s) of olfaction in all horses. Horizontal segments show SD. The mean for dry common sage differs significantly (*p* < 0.05) from those for dry common yarrow, wet common yarrow, wet common chamomile, wet common nettle and wet-sweetened diets apart from the common sage.

The horses studied consumed all the feeds on offer. The effect of the experimental condition (feed presentation_herb) on all the variables studied was statistically highly significant (*p* < 0.001). The mean time of olfaction was the longest in the case of the dry common sage (29.9 ± 35.3 s; Figure 1). It was statistically longer (*p* < 0.05) than that of smelling the dry common yarrow (20.0% of dry diets), wet common yarrow, common chamomile and common nettle (50.0% of wet diets), and 83.3% of the wet-sweetened diets. Moreover, in the case of the wet and wet-sweetened common sage, a non-significant tendency towards a longer time of smelling was noticed.

The horses smelled other dry and wet feeds briefly before ingesting, whereas the other wet-sweetened diets were not smelled at all. The dry feed (5.7 ± 11.9 s) was smelled six times longer than the wet and wet-sweetened feeds (0.8 ± 1.7 s). The horses did not smell or smelled briefly (less than 1 s) in the case of the control oats presented differently.

**Figure 2:**
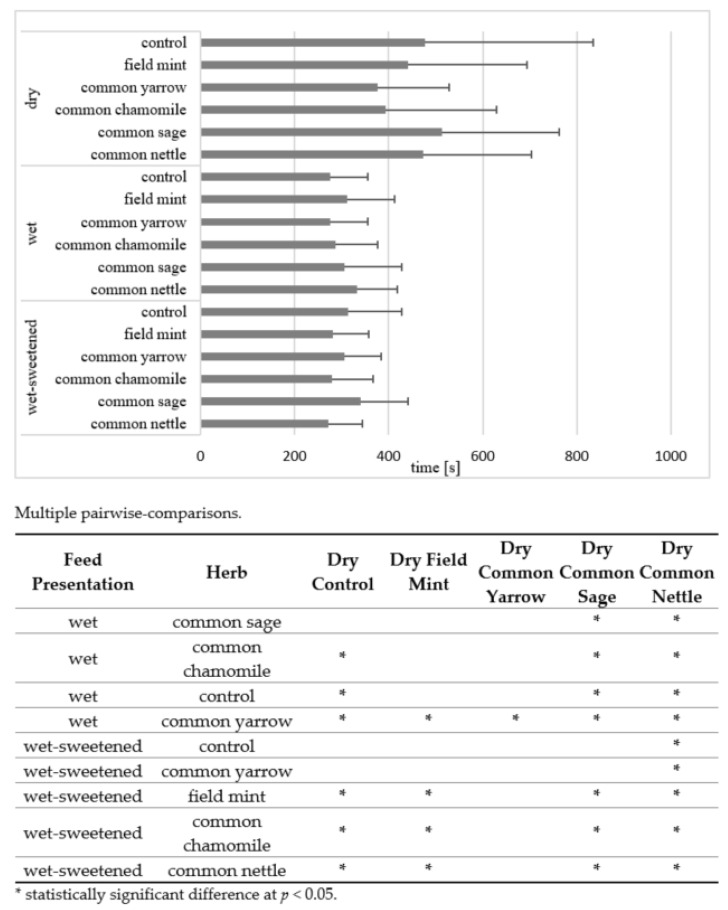
Mean time (s) of consumption in all horses. Horizontal segments show SD. Feed presentations and herbs of no significant differences are omitted within the pairwise-comparisons.

The mean time of consumption was almost 50% longer for dry than wet and wet-sweetened diets (Figure 2). Significant differences (*p* < 0.05) occurred exclusively between dry diets and wet or wet-sweetened diets. The longest time was taken to eat the dry common sage (513.9 ± 248.8 s) and this was 109% longer than the mean of the other dry herbs (245.7 ± 73.6 s). The time was significantly different (*p* < 0.05) from that of most wet and wet-sweetened diets. The next diets eaten in a successively shorter time than the dry common sage were the dry control diet (477.7 ± 357.2 s; 7% shorter), dry common nettle (474.2 ± 229.8 s; 8% shorter) and dry field mint (441.0 ± 253.7 s; 17% shorter), although the differences between these diets were not significant.

**Figure 3:**
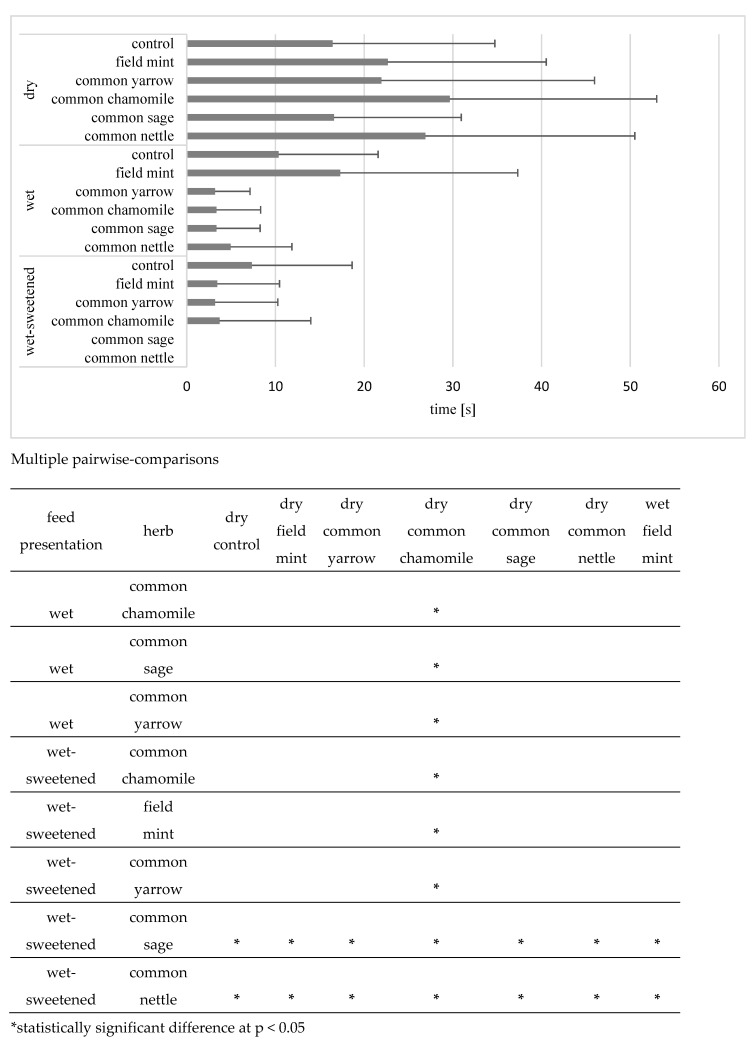
Mean time (s) of intervals in consumption in all horses. Horizontal segments show SD. Feed presentations and herbs of no significant differences are omitted within the pairwise-comparisons.

The longest mean time of intervals in consumption (*p* < 0.05) was recorded for the dry common chamomile (29.7 ± 23.3 s), which was 42% longer than the mean for other dry herbs (20.9 ± 4.4 s): common nettle (26.9 ± 23.6 s), field mint (22.7 ± 17.9 s) and common yarrow (22.0 ± 24.0 s), although the differences between these diets were not significant (*p* > 0.05; Figure 3). The mean time of intervals in the consumption of dry feeds (22.4 ± 5.3 s) was over four times longer than in the case of wet and wet-sweetened feeds (5.0 ± 4.8 s). The time was significantly longer (*p* < 0.05) for three dry diets compared with wet diets, and there were 17 significant differences with wet-sweetened diets. The interval time was also longer for the wet field mint than for the wet-sweetened common nettle and common sage, which were consumed without a break. The time for the control diets of a certain structure did not differ significantly (*p* > 0.05) from that for experimental diets of the same structure.

**Figure 4:**
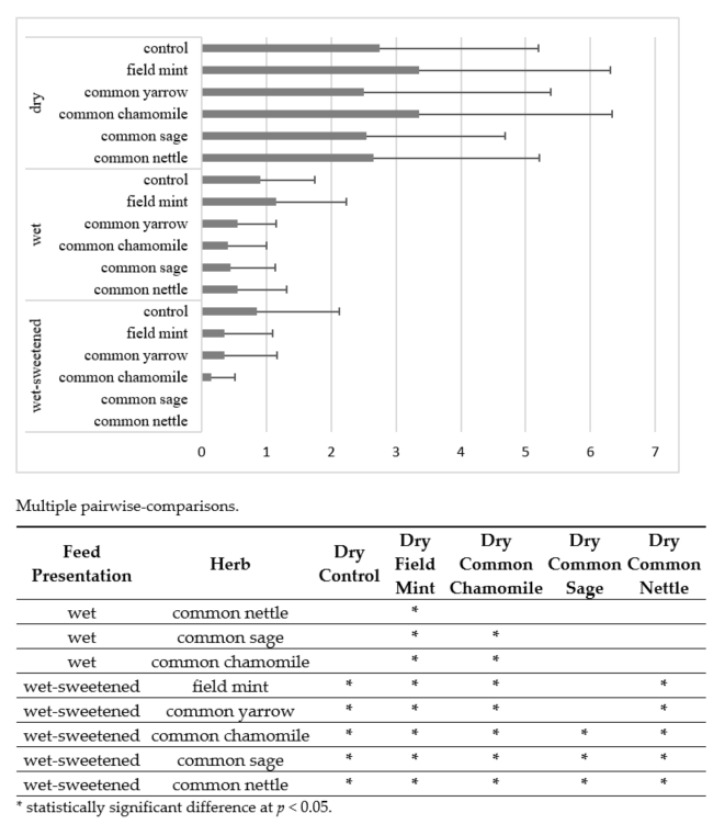
Mean number of intervals in consumption in all horses. Horizontal segments show SD. Feed presentations and herbs of no significant differences are omitted within the pairwise-comparisons.

The mean number of intervals in consumption was higher (*p* < 0.05) for the dry diets compared with wet and wet-sweetened diets in many cases (Figure 4). Most significant differences (*p* < 0.05) occurred between the dry and wet-sweetened diets. The mean number of intervals in the consumption of dry feed (2.86 ± 0.39) was almost 10 times higher than that for wet-sweetened feed (0.28 ± 0.32).

The intervals were frequent, particularly for the dry field mint (3.4 ± 3.0) and common chamomile (3.4 ± 3.0). The number of intervals for the control diets of a certain presentation did not differ significantly (*p* > 0.05) from that for other diets of the same presentation.

**Figure 5:**
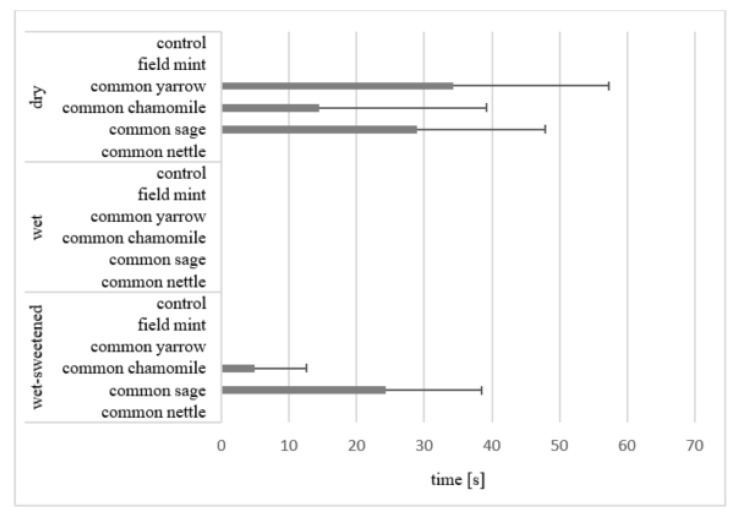
Mean time (s) of drinking water in all horses. Horizontal segments show SD. Mean times for dry common yarrow, dry common sage and wet-sweetened common sage differ significantly (*p* < 0.05) from those for all other diets.

**Figure 6:**
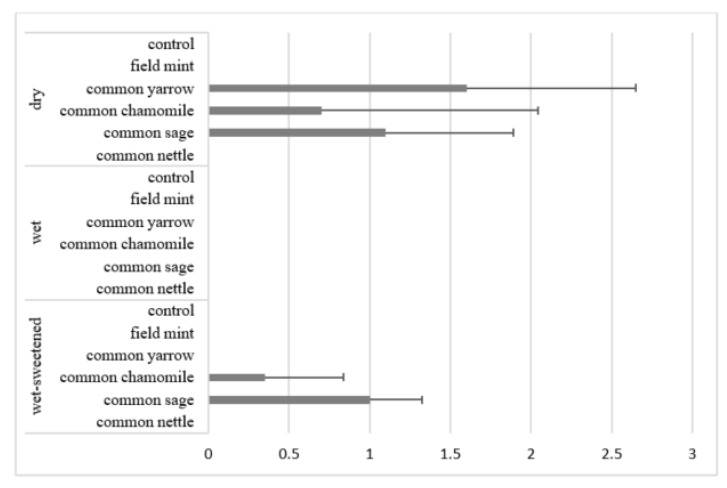
Mean number of times of drinking water in all horses. Horizontal segments show SD. Means for dry common yarrow, dry common sage and wet-sweetened common sage differ significantly (*p* < 0.05) from those for all other diets.

The horses interrupted their consumption to drink water only in the case of five diets: dry common yarrow, dry and wet-sweetened common sage and dry and wet-sweetened common chamomile (Figure 5). The mean time of drinking (*p* < 0.05) was the longest for the dry common yarrow (34.3 ± 23.1 s), i.e., five times longer than the time of other dry and wet-sweetened diets (6.6 ± 10.9). The results of the mean number of times of drinking water were compatible with the mean time of drinking (Figure 6). The mean number of times of drinking was 2.5 times higher for dry feed (0.57 ± 0.68) compared with wet-sweetened feed (0.23 ± 0.40). The horses drank water 1.6 ± 1.0 times in the case of dry common yarrow. When consuming the dry and wet-sweetened common sage, the horses drank water approximately once, whereas in the case of the dry and wet-sweetened common chamomile, the mean number of times of drinking was under one.

**Figure 7:**
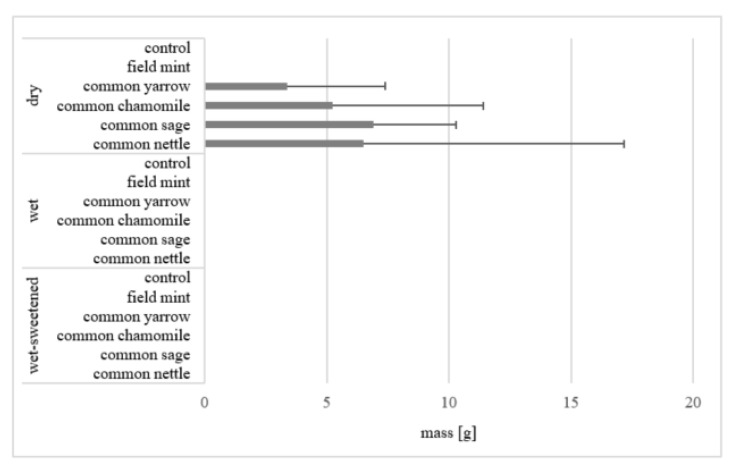
Mean mass (g) of leftovers for all horses. Horizontal segments show SD. Means for dry common sage differ significantly (*p* < 0.05) from those for dry control, dry field mint and all wet and wet-sweetened diets.

The leftovers were recorded in the case of only four diets, which means the mass was small, whereas the SD was usually high (Figure 7). The mean mass of leftovers amounted to 6.9 ± 3.4 g for the dry common sage, 6.5 ± 10.7 g for the dry common nettle, 5.3 ± 6.2 g for the dry common chamomile and 3.4 ± 4.0 g for the dry common yarrow. However, only the mean for dry common sage differed significantly (*p* < 0.05) from those for dry control, dry field mint and all wet and wet-sweetened diets. The mean mass of leftovers in the case of common sage was higher than that of common nettle, common chamomile and common yarrow by 10, 30 and 100%, respectively.

## 4. Discussion

The analysis of the horses’ response to a novel feed revealed that the significant factor which can affect the feed intake is not the herb in the amount offered but diet presentation (dry, wet or wet-sweetened). Considering the methods, it should be pointed out that the response to novelty was assessed in terms of traits directly showing the time of intake (times of olfaction, consumption, times, and numbers of intervals and drinking water) and rejection (the mass of leftovers), whereas other behaviours from the ethogram were not regarded. Another limitation was that the horses studied could have behaved in a different way in the case of an ad libitum oats diet which, however, was not possible to offer taking into consideration the horses’ safety within the short treatment. The horses did not experience unusual hunger because they had been accustomed to the diurnal ration of feed for a long time.

The mean time of olfaction of various feeds indicates that, irrespective of the herbs added, wet-sweetened feeds are usually ingested most willingly, i.e., not smelled before consumption; wet diets are in second place, whereas dry diets are smelled longer. A preference of the smell of a wet feed compared with dry feed was previously reported by Ellis [36]. Considering the time of olfaction, the common sage stands out from other herbs in our study because it was smelled for a longer time than other herbs. The mean mass of leftovers of the dry common sage was higher than that of the dry control diet, which shows that the herb was consumed unwillingly. Common sage is frequently absent in hay offered to horses [37]. Its sharp camphor smell with a citron tone may be novel to the horses and this seems to be a reason for the longer smelling duration [4,38].

Olfaction is the important sense involved in selecting plants ingested by horses, followed by the sense of taste [9]. Smelling a feed before ingesting means that a horse explores the unknown odour of a diet [34]. A familiar odour may increase the acceptance of a novel food [4]. According to Van den Berg and Hinch [11], three to four days are needed to reduce variability in a horse’s response to the novel odour of a feed. In our study, a particular herb was offered three times, within diets of a different presentation, each after 13 days, hence, the feeds were novel for the horses.

Considering the mean time of consumption, the dependence on the presentation of the concentrated feed is clearly visible. The horses consumed wet and wet-sweetened feeds more quickly compared with dry feeds. The dependence is still more distinct for the time and number of intervals in consumption. The wet and wet-sweetened feeds are ingested with fewer breaks. The leftovers found exclusively in the case of the dry diets confirm that the feed of such presentation is ingested less willingly than wet and wet-sweetened feeds. Ellis [36] reported that the voluntary intake of wet and molassed straw is higher than the intake of untreated straw. Adding wet sugar beet pulp to concentrated feed leads to faster intake rates. The author notes that increasing moisture content of short particle feed causes a considerable reduction in chewing times due to a decreased need for saliva production. In turn, the sweetness affects the organoleptic perceptions of horses. Van den Berg et al. [26] found a positive influence of a non-caloric natural sweetener on the diet choice in horses.

Assuming that a palatable feed is ingested quickly and almost without breaks, it can be inferred that the concentrated feed offered to horses should be wetted or wetted and sweetened. However, the fact that wet feeds do not allow animals to separate feed ingredients should also be considered. Dry forages, such as hay, provide this possibility, along with playing with the feed.

The time of consuming feed and the time and number of intervals in consumption of the herbs studied show that the effect of particular herbs did not differ. The herbal supplement did not elicit any significant changes in the horses’ behaviour compared with control diets. The lack of significant differences in the response to the addition of different herbs indicates that the horses identified the herbs in the amount offered weakly and their feeding behaviour was indifferent to the herb species. It may be speculated that properties of the herbs studied, except the common sage, were not sufficiently distinct to affect the horses’ sensory experience during feed intake.

Times of consuming feeds are frequently used to determine the palatability of the flavours tested [2,10]. Figueroa et al. [39], for example, considered the consumption time per approaches in pigs and rats, whereas Khelil-Arfa et al. [21] assessed the amount of concentrate consumed during a 2 min offering in horses. The amount of food offered in our study was constant, hence, the consumption time, not speed, could be considered. Earlier findings indicate that various plants added to feed may accelerate consumption or, on the contrary, inhibit it or cause rejection behaviour from the horses [6,34]. Horses may feel a need to consume certain herbs with wide therapeutic properties when their body faces different afflictions. However, horses find it difficult to associate a feed with its nutritional consequences because of long gut-transit times and other feeds offered simultaneously [40]. Hence, this motivator could not have acted in our study, in which the horses were offered each kind of feed only once and could rely only on the sight, smell, taste and touch of the feed texture during ingestion.

Our results suggest that the amount of the herbs was probably too little to show differences in the response to novelty and the effect should be studied in the future with a higher quantity of the herbs than the producer’s recommendation. However, it cannot be excluded that an enhancement of the herb content in the feed may limit the intake instead of increasing it. The herbal addition should be increased with caution because some herbs may disturb the animal’s health. The present study considers the effects of only five herbs offered within novel diets on some aspects of horses’ feeding behaviour. It can be suggested that many other herbs warrant investigation in this regard.

Interestingly, the horses interrupted their consumption to drink water only in the case of the dry and wet-sweetened feeds. The wet diets did not elicit any need to drink water. Firstly, these results may be a consequence of the starch in the dry oats which needs more saliva to form a moist bolus for swallowing. Secondly, the sweet taste leads to a need to drink water. It is known that horses usually drink during or after a feeding bout when they have free access to water [4]. According to Murphy et al. [41], water consumption in horses may be enhanced by offering a variety of flavours. Manufacturers supplement horse feeds in an attempt to encourage water drinking, for example, to avoid dehydration under specific conditions [21]. Our results show that the horses interrupted their consumption to drink water in the case of dry common yarrow, dry and wet-sweetened common sage, and common chamomile. The common yarrow traditionally used in human treatment has a nice meadow odour; the common sage, as has been mentioned, has a camphor smell with a citron tone, whereas the common chamomile has a strong herbal odour [4,28,29,38,42]. All these three herbs have a slightly bitter taste and perhaps this is the factor which elicits the horses’ need to drink water during feed consumption.

## 5. Conclusions

The results of the horses’ response to a novel feed indicate that the significant factor which can affect their willingness to consume the feed is not the herb in small amounts but feed presentation (dry, wet or wet-sweetened). The properties of the field mint, common yarrow, common chamomile and common nettle do not disturb consumption and only the odour of the common sage may delay intake. Wetting or wetting and sweetening the diet positively affects the horses’ willingness to consume. In spite of the fact that wet and wet-sweetened diet presentations may be novel to horses, they increase the feed palatability. Wetting and sweetening feed is an undemanding procedure and, thus, can easily be applied when preparing diets for horses.

## Figures and Tables

**Table 1:** Experimental schedule in horse groups.

Consecutive Days	Feed Presentation in HORSE GROUPS			Herbal Addition
	1 Group *n* = 7	2 Group *n* = 7	3 Group *n* = 6	
First stage				
1	dry	wet	wet-sweetened	control
2	dry	wet	wet-sweetened	field mint
3	dry	wet	wet-sweetened	common yarrow
4	dry	wet	wet-sweetened	common chamomile
5	dry	wet	wet-sweetened	common sage
6	dry	wet	wet-sweetened	common nettle
7–13	Interval			
Second stage				
14	wet	wet-sweetened	dry	control
15	wet	wet-sweetened	dry	common yarrow
16	wet	wet-sweetened	dry	common chamomile
17	wet	wet-sweetened	dry	common sage
18	wet	wet-sweetened	dry	common nettle
19	wet	wet-sweetened	dry	field mint
20–26	Interval			
Third stage				
27	wet-sweetened	dry	wet	control
28	wet-sweetened	dry	wet	common chamomile
29	wet-sweetened	dry	wet	common sage
30	wet-sweetened	dry	wet	common nettle
31	wet-sweetened	dry	wet	field mint
32	wet-sweetened	dry	wet	common yarrow

*n*—number of horses.

## Data Availability

The data presented in this study are available on request from the corresponding author.
